# Co-occurring Symptoms of ADHD, Autism, Oppositional Defiant Disorder, and Academic Impairment in Referred and Community Children

**DOI:** 10.1007/s10862-026-10317-0

**Published:** 2026-09-12

**Authors:** Susan D. Mayes, Mara Villalongo Andino, Richard E. Mattison, Daniel A. Waschbusch

**Affiliations:** 1 Department of Psychiatry and Behavioral Health, Penn State College of Medicine, 500 University Dr, Hershey, PA 17033 USA; 2 Department of Psychology, Virginia Tech, Blacksburg, VA USA

## Abstract

Our study is the first to compare in referred and population-based samples the co-occurrence of four common problems for which children are referred for psychological evaluations (ADHD, autism, oppositional defiant disorder/ODD, and academic impairment). Youth were rated by their mothers on the Pediatric Behavior Scale, assessing symptoms of ADHD, autism, ODD, and academic impairment. The 1,601 referred children (6–16 years) were diagnosed with autism and/or ADHD. The population-based samples comprised 665 children 6–12 years and 376 re-evaluated a mean of 8 years later. All children had an IQ *≥* 70. Co-occurrence of ADHD, autism, ODD, and academic impairment symptoms was high in the referred and community samples. In the referred sample, 90% had symptoms of two or more of these diagnoses. In the community samples, the risk of two or more symptom elevations was as high as the risk of having only one. Overall, most youth with autism had ADHD-Combined and ODD, most youth with ADHD-Combined had ODD, and most youth with ADHD-Combined and ADHD-Inattentive had academic impairment. Ratings of inattention were more strongly associated with academic impairment than with autism and ODD, whereas hyperactive/impulsive ratings were associated with ODD. Across samples, inattention was a significant and unique correlate of academic impairment ratings and low reading and math achievement test scores, whereas hyperactivity/impulsivity was a unique correlate of ODD. Given high co-occurrence across clinical and community contexts, evaluations should routinely assess ADHD, autism, ODD, and academic impairment together so that treatable conditions that compound each other are identified and addressed.

Comorbid psychopathology is common in children with psychological disorders including ADHD, autism, and oppositional defiant disorder (ODD) (Hawes et al., 2023; Joshi et al., 2010; Kadesjö & Gillberg, 2001; Leyfer et al., 2006; Simonoff et al., 2008; Willcutt et al., 2012). Our study focuses on four of the most common childhood disorders for which children are referred for psychological evaluations: ADHD, autism, ODD, and academic impairment. The study is the first to determine the comparative co-occurrence of symptoms of these conditions in both large referred and population-based child and adolescent samples. Findings will have important implications for understanding how these concerns cluster together across both clinical and community settings, which is essential for accurately interpreting symptoms, planning intervention, and guiding future research.

## ADHD, Autism, and ODD

Most referred children and adolescents with autism have ADHD with percentages of 76% (Joshi et al., 2017), 81% (Lecavalier et al., 2019), 80% for ADHD-Combined (ADHD-C) and 9% for ADHD-Inattentive (ADHD-I) presentation (Mayes et al., 2024), and 67% using parent ratings and 71% using teacher ratings, with much higher rates based on either informant (Kaat et al., 2013). A meta-analysis of 56 studies combining national and international community and clinical samples of autistic children and adults in which individuals were evaluated for ADHD using clinical interviews or questionnaires, yielded a mean ADHD prevalence rate of 48% for children 6–17 years (Rong et al., 2021).

In contrast to the high prevalence of ADHD in referred autistic children, the prevalence of autism in children with ADHD was lower in a study in which all children were evaluated using parent interviews (Joshi et al., 2017). Autism symptoms are more common in ADHD-C than in ADHD-I (Gadow et al., 2006). Importantly, a diagnosis of autism may be delayed in children with ADHD because ADHD symptoms may obscure or overshadow autism symptoms (Hartley & Sikora, 2009; Rong et al., 2021). Children with autism initially diagnosed with ADHD were diagnosed with autism an average of 2 years later than children with autism and no ADHD (Kentrou et al., 2019). Similarly, for children with autism who were originally diagnosed with only ADHD, the autism diagnosis was made approximately 3 years later than children who were diagnosed with ADHD at the same time as or after the autism diagnosis (Miodovnik et al., 2015). The mean age at diagnosis for children with only autism was approximately 2.5 years, whereas children who had autism and ADHD were diagnosed with autism on average after age 6 (Stevens et al., 2016).

Research studies and systematic reviews investigating prevalence rates of ADHD and autism and their co-occurrence are limited by the fact that not all children have been assessed for autism or ADHD using standardized rating scales, interviews, or clinical evaluations, resulting in an underestimation of diagnoses and prevalence rates. In many studies, parents are simply asked “Has your child ever been diagnosed with ADHD or autism?” or charts are reviewed to ascertain whether or not a child has been given a diagnosis of ADHD or autism. In two large surveys, only 50% and 47% of children who were significantly elevated on parent ADHD ratings had been evaluated and professionally diagnosed with ADHD (Barkley, 2013; Burns & Becker, 2021). Therefore, ADHD was potentially missed in many of these children. Further, the DSM-IV did not permit a diagnosis of ADHD with autism (which the DSM-5 now allows). As a result, ADHD was underdiagnosed in autism. Further, in studies of children with ADHD and children with autism, research participants are often not evaluated for possible co-occurring autism and ADHD, which would affect the interpretation of research findings (e.g., study results may be attributed to ADHD when they are instead related to ADHD’s association with autism and vice versa).

Averaging across studies, approximately half of children with ADHD-C have ODD, whereas percentages are lower in ADHD-I (e.g., Abikoff et al., 2004; Connor et al., 2010; Efron & Sciberras, 2010; Faraone et al., 1998; King & Waschbusch, 2010; MTA Cooperative Group, 1999; Waschbusch, 2002). In contrast to extensive and relatively consistent research findings for ODD in ADHD, research on ODD in autism is markedly inconsistent. A review of nine studies using structured diagnostic interviews in autistic children and adolescents, ODD prevalence rates ranged from 4% to 75% (van Steensel et al., 2013).

## Academic Impairment

Studies of community and clinical youth have shown an association between ADHD and learning disabilities, low grades, and/or low achievement test scores (Arnold et al., 2020; Goldston et al., 2007; Willcutt & Pennington, 2000; Willcutt et al., 2013). Research indicates that the association is primarily explained by the attention deficit and not hyperactivity/impulsivity (Maughan et al., 2003). Several studies investigating the relationship between behavior problems (including ODD) and academic impairment show a nonsignificant relationship when attention problems are controlled (Barriga et al., 2002; Fergusson & Horwood, 1995; Fergusson et al., 1993; Maguin & Loeber, 1996; Mattison & Mayes, 2012). Further, children with ODD without ADHD have fewer learning problems than children with ADHD-C and ADHD-I (Mayes & Calhoun, 2006). Research comparing learning problems in youth with ADHD versus autism is scarce, as is research examining relationships between academic impairment and behavior problems in autism. One study found children with ADHD had a higher learning disability rate than autistic children (Mayes & Calhoun, 2007). Another study reported that behavior problems were not associated with academic achievement in 30 autistic children (Estes et al., 2011).

## Purpose

The present study compares co-occurrence patterns for four of the most common child diagnostic clinic referral concerns (ADHD, autism, ODD, and academic impairment) across a large referred ADHD/autism sample and population-based child and adolescent samples using the same symptom measure, which has not been done in previous studies. The goal of our study is to show how commonly these domains overlap across clinical and community contexts and how inattention and hyperactivity/impulsivity relate differentially to autism, ODD, and academic impairment. This comparative focus provides clinically useful information while underscoring the need for comprehensive assessment when any of these problems is present. Our study aims to (1) determine the prevalence and co-occurrence of ADHD-C, ADHD-I, autism, ODD, and academic impairment symptoms in referred children with ADHD and/or autism and in community children and adolescents and (2) examine associations between symptom ratings of inattention, hyperactivity/impulsivity, autism, ODD, and academic impairment that may underly and explain co-occurrence.

## Methods

### Samples

The referred sample comprised 1,601 children with autism and/or ADHD, 6–16 years of age, and IQ *≥* 70 whose mothers rated their children on the Pediatric Behavior Scale (PBS; Lindgren & Koeppl, 1987). All children were referred to a department of psychiatry and behavioral health diagnostic clinic because of behavior problems and/or possible autism or ADHD. The mean sample age was 8.9 years (*SD* 2.6), and the mean IQ was 102.6 (*SD* 15.3, range 70–149). In all, 73.6% were male, 39.8% had a parent with a professional or managerial occupation, and 92.1% were White. Reading and math achievement test data were available for 967 of these children. Demographic characteristics of children with achievement data were similar to the total sample (6–16 years, mean 8.9 years, *SD* 2.4; IQ 70–145, mean 102.7, *SD* 15.2; 75.7% male; 40.8% parent professional or managerial occupation; and 92.9% White).

The population-based samples comprised 665 elementary school children evaluated at baseline and 376 re-evaluated a mean of 8 years later who participated in a population-based epidemiologic study of sleep disorders (Bixler et al., 2009). Reading and math achievement test scores were available for 583 children at baseline. The baseline elementary school sample was 6–12 years of age (mean age 8.7, *SD* 1.7). IQs ranged from 71 to 147 (*M* 106.3, *SD* 13.1). In all, 52.6% were male, 80.5% were white, and 48.9% had a parent with a professional or managerial occupation. The follow-up sample had a mean age of 16.4 years (*SD* 2.2, range 12–23). IQs ranged from 78 to 147 (*M* 107.8, *SD* 13.1). In all, 54.3% were male, 82.2% were white, and 50.3% had a parent with a professional or managerial occupation. The baseline and follow-up samples did not differ significantly in sex, race, or parent occupation (χ^2^ = 0.2–0.5, *p* > .05).

### Instruments

Symptoms of ADHD, autism, ODD, and academic impairment were assessed with the PBS, on which items were rated by mothers on a 4-point scale from 0 (“almost never or not at all”) to 3 (“very often”) a problem during the past 2 months. The PBS corresponds well with established measures of psychopathology and has been used to diagnose and differentiate psychological problems in multiple studies (Calhoun et al. 2017; Conrad et al. 2010; Lindgren & Koeppl 1987; Mattison & Mayes 2012; Mayes et al. 2009, 2024; Nichols et al. 2000; Waxmonsky et al. 2017; Wolraich et al. 1994). PBS subscales used in the present study and their item composition were attention deficit (short attention span, easily distracted, shifts quickly from one activity to another, fails to finish things he/she starts), hyperactivity/impulsivity (hyperactive, restless/cannot sit still, squirms/fidgets, constantly in motion, always running about or climbing on things, acts without thinking first, cannot stand waiting, interrupts, grabs for things/into everything, rushes into danger without thinking about getting hurt), hyperactivity, oppositional defiant disorder (disobedient, argues, uncooperative, defiant, irritable, overacts to minor problems, explosive, loses temper/tantrums), autism (withdrawn/spends a lot of time alone, odd movements such as hand flapping and toe walking, talks or thinks about the same thing over and over, repeats certain actions over and over, upset by change/insists on doing things the same way every time), and academic impairment (difficulty learning even when tries hard, underachieving, low grades, and trouble with reading, writing, or math).

The Wechsler Individual Achievement Test (WIAT or WIAT-II) Word Reading and Numerical Operations subtests were administered in the referred sample. Children in the community sample were evaluated with the Wide Range Achievement Test-Third Edition Reading and Arithmetic subtests.

### Referred Sample Diagnostic Procedure

Children in the referred sample all had a diagnosis of ADHD and/or autism determined by a multi-source and instrument diagnostic evaluation by a licensed PhD psychologist that included a diagnostic interview with the parents, standardized parent and teacher rating scales, administration of psychological tests, and clinical observations of the child during the evaluation. Children with autism had a DSM-IV or DSM-5 diagnosis of autism (i.e., autistic disorder, Asperger’s disorder, or autism spectrum disorder) and a score in the autism range on the Checklist for Autism Spectrum Disorder (CASD; Mayes, 2012). The CASD is an autism diagnostic instrument completed by a clinician based on a diagnostic interview with the parents, teacher completion of the CASD, and clinical observations of the child during the evaluation. Studies show the CASD has strong validity and differentiates children with autism from children with other disorders without autism (Mayes, 2012; Mayes et al., 2012a, 2012b; Murray et al., 2011; Tierney et al., 2015). Children with ADHD had a DSM-IV or DSM-5 (whichever version was current when the child was evaluated) diagnosis of ADHD made by a licensed PhD psychologist and fulfilled the following criteria: symptoms of ADHD observed during psychological testing and PBS item ratings of short attention span and/or distractible as often or very often a problem by at least two raters (mother, father, and/or teacher). Children were classified with ADHD-C if two or three of the mother, father, and teacher ratings on the combined hyperactive-impulsive PBS items were often or very often a problem. Children were classified with ADHD-I if the combined hyperactive-impulsive ratings were below this threshold. Multiple raters were used to fulfill the DSM criterion of cross-setting ADHD symptoms. Children were identified with ODD if their mean score on the PBS ODD symptom scale rated by mothers was more than sometimes a problem. This is consistent with the DSM stipulation that ODD should be diagnosed if symptoms are reported in only one setting, which according to the DSM is usually the home environment. Academic functioning was assessed in two ways. Children were designated as having academic impairment if their mean PBS academic impairment score was greater than sometimes a problem. Low achievement in reading and math was defined as a standardized achievement test score *≤* 1 *SD* below the normal mean.

### Community Sample Symptom Groups

Unlike the referred sample, children and adolescents in the population-based sample did not undergo individual diagnostic evaluations by a PhD psychologist. Instead, children and adolescents were assigned to symptom groups based on maternal PBS symptom ratings. Children and adolescents with symptoms of ADHD-C, ADHD-I, autism, ODD, and academic impairment were those youth whose maternal ratings on the corresponding subscales were greater than sometimes a problem.

### Data Analyses

Co-occurrence percentages for ADHD-C, ADHD-I, autism, ODD, and academic impairment symptoms for the referred and community samples were graphed and presented descriptively for each group. ANOVA and Cohen’s *d* were used to assess the statistical and clinical significance of differences in ODD and academic impairment scores between diagnostic groups in the referred sample. Differences in frequencies of elevated inattentive, hyperactive/impulsive, autism, and ODD ratings between children with and without low achievement (reading and math achievement test scores *≤* 85) were examined with chi square and phi. A cut point of 85 was chosen because it is a conventional and statistically determined value (i.e., 1 *SD* below the normal mean) that is objective and empirically significant. Associations between ratings of inattention, hyperactivity/impulsivity, autism, ODD, and academic impairment were investigated with Pearson correlations and explained variance. Unique and significant correlates of ODD and academic impairment ratings and reading and math achievement test scores were determined with stepwise linear regression analysis.

## Results

### Co-Occurrence of Symptoms

#### Referred sample

Co-occurrence was more likely than not in the referred sample. For these children (all of whom had ADHD or autism or both), 89.8% had symptoms of two or more diagnoses (autism, ADHD, ODD, and/or academic impairment). In total, 34.3% had symptoms of two diagnoses, 37.8% had three, and 17.7% had four. Only 10.2% had ADHD or autism alone.

#### Community samples

Co-occurrence of elevated maternal ratings (greater than sometimes a problem) on the four symptom subscales (ADHD-C or ADHD-I, autism, ODD, and academic impairment) was also high in the child and adolescent community samples. In the child sample, 60.2% were not elevated on any of the four symptom subscales, whereas 19.5% were elevated in only one area and 20.3% were elevated on two or more subscales (13.1% on two, 6.6% on three, and 0.6% on all four). Results were similar in the adolescent community sample (71.5% not elevated on any subscale, 15.4% elevated on only one, 8.8% on two, 4.0% on three, and 0.3% on all four subscales).

### Prevalence Rates and Mean Scores

#### Referred ADHD/autism sample

**Fig. 1:**
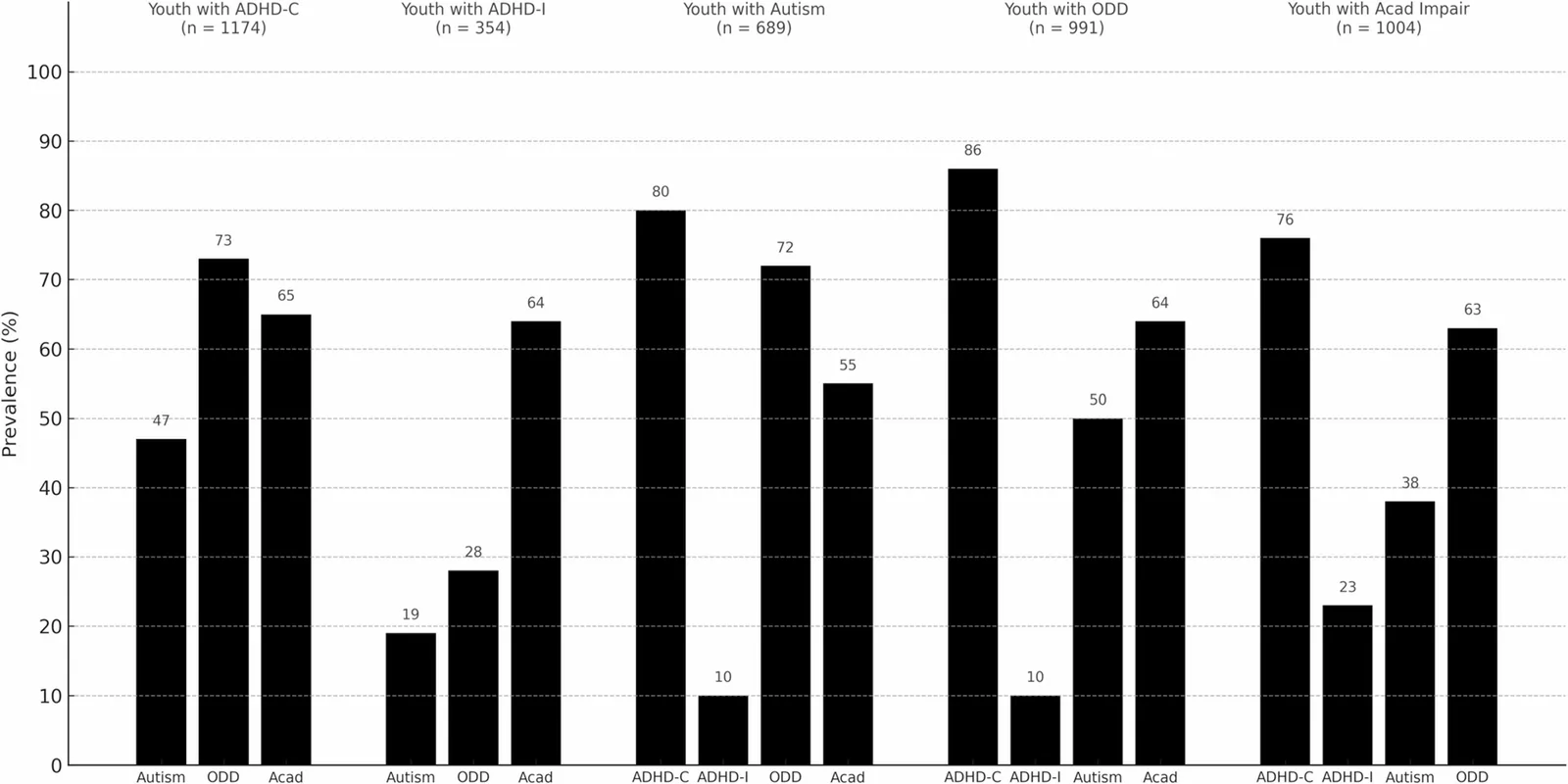
Percentages for co-occurrence of symptoms of ADHD-combined, ADHD-inattentive, autism, oppositional defiant disorder, and academic impairment in referred youth (*N* = 1,601) Note. ODD = oppositional defiant disorder, Acad and Acad Impair = elevated academic impairment ratings

As shown in Fig. 1, high symptom prevalence rates were found in many domains. For youth with ADHD-C, 73% had ODD symptoms and the majority of youth with ADHD-C and ADHD-I had elevated academic impairment ratings. For youth with autism, 80% had ADHD-C and 72% had ODD. Most (86%) youth with ODD had ADHD-C, and 99% of youth with academic impairment had ADHD (ADHD-C or ADHD-I).

Youth with ADHD-C plus autism followed by ADHD-C only had significantly higher ODD rating scale scores than all other groups (autism only, ADHD-I plus autism, and ADHD-I only), *F* = 89.4, Bonferroni *p* < .05, *d* = 0.4–1.2. Academic impairment ratings were significantly higher for youth with ADHD-I only and ADHD-C only than for autism only, ADHD-I plus autism, and ADHD-C plus autism (*F* = 27.9, *p* < .001, *d* = 0.2–1.2).

Frequencies of hyperactivity/impulsivity, ODD, and autism symptom ratings greater than sometimes a problem did not differ significantly (*χ*^2^ = 1.3–1.6, *ϕ* = 0.1, *p* > .05) between children with and without reading achievement tests scores *≤* 85, whereas inattention frequencies were significantly higher in the low reading group (*χ*^2^ = 4.0, *ϕ* = 0.1, *p* < .05). Frequencies of hyperactivity/impulsivity and ODD symptom scores greater than sometimes a problem did not differ significantly between children with and without math achievement test scores *≤* 85 (*χ*^2^ = 3.1 and 3.0, *ϕ* = 0.1, *p* > .05), whereas inattention and autism frequencies were significantly higher in the low math group (*χ*^2^ = 4.5 and 5.5, *ϕ* = 0.1, *p* < .05).

#### Community child sample

**Fig. 2:**
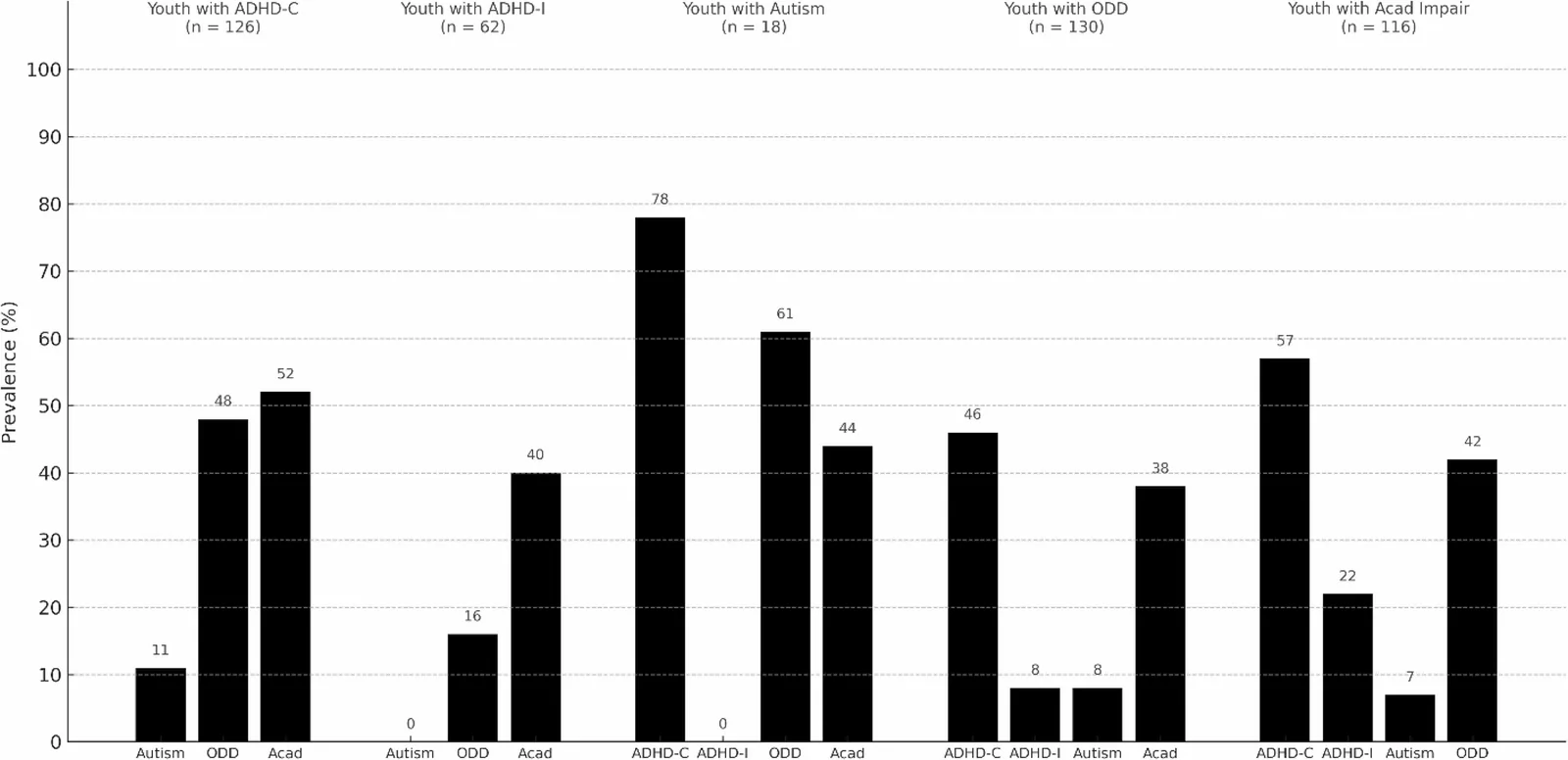
Percentages for co-occurrence of elevated maternal ratings (greater than sometimes a problem) of ADHD, autism, oppositional defiant disorder, and academic impairment symptoms in community children (*N* = 665) Note. ODD = oppositional defiant disorder, Acad and Acad Impair = academic impairment

For community children with elevated symptom ratings of ADHD-C (greater than sometimes a problem), approximately half had elevated academic impairment and ODD ratings (Fig. 2). For youth with elevated ADHD-I ratings, 40% had elevated academic impairment ratings. Most youth with elevated autism ratings had elevated ADHD-C and ODD ratings. Almost half of youth with elevated ODD ratings had elevated ADHD-C ratings. Last, 79% of youth with elevated academic impairment ratings had symptoms of ADHD (ADHD-C or ADHD-I).

Frequencies of ODD and autism symptom ratings greater than sometimes a problem did not differ significantly (*χ*^2^ = 2.3 and 1.1, *ϕ* = 0.1 and 0.0, *p* > .05) between children with and without reading achievement test scores *≤* 85, whereas inattention and hyperactivity/impulsivity frequencies were significantly higher in the low reading achievement group (*χ*^2^ = 16.0 and 11.2, *ϕ* = 0.2 and 0.1, *p* < .05). Frequencies of autism symptom scores greater than sometimes a problem did not differ significantly between children with and without math achievement test scores *≤* 85 (*χ*^2^ = 0.1, *ϕ* = 0.0, *p* > .05), whereas inattention, hyperactivity/impulsivity, and ODD frequencies were significantly higher in the low math achievement group (*χ*^2^ = 33.5, 9.6, and 4.7, *ϕ* = 0.1–0.2, *p* < .05).

#### Community adolescent sample

**Fig. 3:**
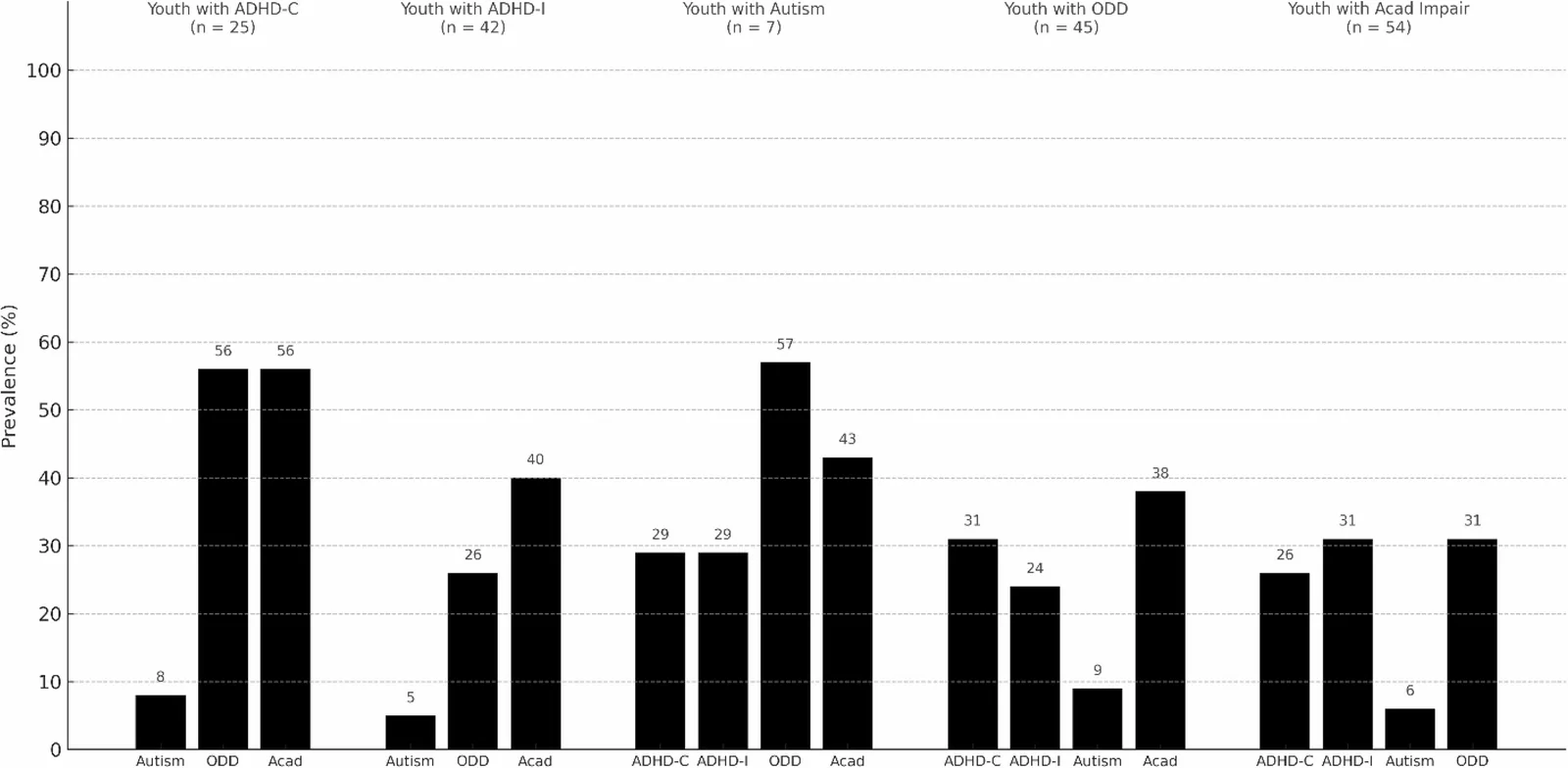
Percentages for co-occurrence of elevated maternal ratings (greater than sometimes a problem) of ADHD, autism, oppositional defiant disorder, and academic impairment symptoms in community adolescents (*N* = 376) Note. ODD = oppositional defiant disorder, Acad and Acad Impair = academic impairment

For adolescents with elevated symptom ratings of ADHD-C, 56% had ODD and 56% had elevated academic impairment ratings (Fig. 3). For adolescents with elevated ADHD-I ratings, 40% had elevated academic impairment ratings. ODD symptoms were present in 57% of adolescents with elevated autism ratings. For adolescents with elevated ODD and elevated academic impairment ratings, approximately half had elevated ADHD ratings (ADHD-C or ADHD-I).

### Correlations and Regression Analyses

Because the diagnostic symptom groups were not mutually exclusive (except for ADHD-C and ADHD-I) and because comorbidity and overlapping symptoms were common, correlational analyses were conducted to determine which specific symptoms were most strongly associated with co-occurring symptoms (Table 1). In all three samples (referred, child community, and adolescent community), inattention was more strongly associated with academic impairment ratings than with autism and ODD ratings, whereas hyperactivity/impulsivity was more strongly associated with ODD than with autism and academic impairment ratings. The only large correlations (> 0.50, explained variance 28.1% − 44.9%) were between ratings of inattention and academic impairment and between hyperactivity/impulsivity and ODD.

**Table 1:** Pearson correlations between pediatric behavior scale symptom ratings

	Inattention	Hyperactive/Impulsive	Autism	ODD
ADHD/Autism sample				
Autism	.16^*^	.32*		
ODD	.28^*^	.53*	.33^*^	
Academic Impairment	.38*	.08*	-.01	.08*
Child community sample				
Autism	.42*	.48*		
ODD	.49*	.59*	.43*	
Academic Impairment	.67*	.48*	.28*	.38*
Adolescent community sample				
Autism	.32*	.35*		
ODD	.50*	.62*	.33*	
Academic Impairment	.67*	.45*	.39*	.44*

ODD = oppositional defiant disorder

^*^p < .01

Sample sizes: ADHD/Autism Sample (n = 1,601), Child Community Sample (n = 665), and Adolescent Community Sample (n = 376)

Regression analyses supported these findings. In the referred sample, only inattention (and not hyperactivity/impulsivity, ODD, and autism ratings) was a unique and significant correlate of academic impairment ratings (*R* = .38, *F* = 263.4, *p* < .001). Inattention was the only significant correlate of lower reading and math standard scores (*R* = .09, *F* = 8.6, *p* = .004 and *R* = .14, *F* = 19.3, *p* < .001). Hyperactivity/impulsivity correlated strongly with ODD (*R* = .53, *F* = 620.8, *p* < .001), while inattention, autism, and academic impairment ratings contributed < 3% more to explained variance. Results for the community child sample were similar. Inattention was a unique correlate of academic impairment ratings (*R* = .67, *F* = 539.6, *p* < .001) and hyperactivity/impulsivity, ODD, and autism ratings were not. Inattention was the only significant correlate of lower reading and math standard scores (*R* = .24, *F* = 36.0, *p* < .001 and *R* = .28, *F* = 48.6, *p* < .001). Hyperactivity/impulsivity correlated strongly with ODD (*R* = .59, *F* = 348.1, *p* < .001), and inattention, autism, and academic impairment ratings contributed < 4% more to explained variance. Results for the community adolescent sample were consistent with these findings. Inattention uniquely correlated with academic impairment ratings (*R* = .67, *F* = 306.2, *p* < .001), whereas hyperactivity/impulsivity, ODD, and autism ratings increased explained variance by < 4%. Hyperactivity/impulsivity correlated strongly with ODD (*R* = .62, *F* = 231.5, *p* < .001), and inattention, autism, and academic impairment ratings contributed < 4% more to explained variance.

## Discussion

Despite decades of research on ADHD, autism, and ODD, clinical assessment and research models often continue to treat these conditions separately. This disconnected approach hides how these disorders actually appear in children; that is, as overlapping symptom clusters that together influence academic and behavioral outcomes. The present study tackled this gap by measuring the co-occurrence of four of the most common referral concerns in both referred and community youth. Findings suggest that, although these conditions can occur independently, overlapping symptom profiles across these domains are common and clinically relevant in both clinical and community settings.

Co-occurrence of ADHD, autism, ODD, and academic impairment symptoms was high in the referred ADHD/autism and community samples. In the referred sample, 90% had two or more of these diagnoses. In the community samples, the risk of two or more symptom elevations was as high as the risk of having only one. Importantly, the high rates of co-occurrence seen in the community samples show that these patterns are not just due to referral bias but represent the wider population of children seen in primary care, schools, and community mental health settings. Therefore, it is critical for clinicians and researchers not to stop when one diagnosis is made and, instead, to rule in or out common co-occurring problems. Such comorbidities negatively affect functioning, require additional intervention, and need to be considered when interpreting research findings that could be confounded by comorbid conditions.

In the referred ADHD/autism sample, ADHD-C was found in most children with autism (80%) and an additional 10% had ADHD-I. Further, 47% with ADHD-C had autism, as did 19% with ADHD-I. Autism symptoms were rare (< 3%) in the community samples, but when present in the child sample, 78% also had ADHD-C symptoms, similar to the pattern found in the referred sample. ADHD-C was more strongly associated with autism than was ADHD-I. ADHD is often not considered or recognized in youth diagnosed with autism, which is a huge oversight that prevents these children from receiving effective ADHD interventions that can improve functioning. The DSM-IV did not permit a diagnosis of ADHD with autism (which the DSM-5 now allows), resulting in the underdiagnosis and treatment of ADHD. Conversely, a smaller but not insignificant percentage of referred children with ADHD have autism, which unfortunately is also often missed and untreated (Hartley & Sikora, 2009; Kentrou et al., 2019; Miodovnik et al., 2015; Rong et al., 2021; Stevens et al., 2016). These diagnostic delays represent missed windows for early, evidence-based intervention during periods of heightened neurodevelopmental plasticity.

In the referred ADHD/autism sample, youth with ADHD-C and youth with autism had very high and similar rates of ODD symptoms (73% and 72%). Almost all youth with ODD symptoms had ADHD-C (86%) and half had autism. The connection between ADHD-C and ODD has long been recognized (Abikoff et al., 2004; Connor et al., 2010; Efron & Sciberras, 2010; Faraone et al., 1998; King & Waschbusch, 2010; MTA Cooperative Group, 1999), but ODD is often neglected in autism assessments and in research, even though ODD symptoms are common in autism. In fact, a recent review of comorbidity in autism did not even mention ODD whereas it was noted that ODD commonly occurs with ADHD, conduct disorder, anxiety, and depression (Hawes et al., 2023). Further, the DSM-5 does not mention ODD as a comorbid condition in autism, whereas other conditions are noted.

Mean maternal ratings of academic impairment (difficulty learning even when tries hard, underachieving, low grades, and trouble with reading, writing, or math) were elevated (more than sometimes a problem) in the majority of referred children and almost half of community children and adolescents with symptoms of ADHD-C, ADHD-I, autism, and ODD. This may reflect interference from these diagnoses with learning and day-to-day functioning in the classroom, as well as the presence of inattentive symptoms associated with these diagnoses. Academic impairment ratings were significantly higher for youth with ADHD-I only and ADHD-C only than for autism only, ADHD-I plus autism, and ADHD-C plus autism. Therefore, adding autism to ADHD decreased academic impairment ratings. Further, academic impairment ratings were more highly correlated with inattention ratings than with ODD, hyperactive/impulsive, and autism ratings in all three samples. Together, these findings support the differentiation of symptom effects; inattention primarily appears to negatively impact academic learning, whereas hyperactive/impulsive symptoms are more closely associated with the oppositional behavior and emotional dysregulation that characterizes ODD.

Analyses of individually administered reading and math achievement test scores showed that only the frequency of inattention ratings greater than sometimes a problem was significantly higher in referred youth with both reading and math achievement test scores *≤* 85 (vs. > 85), whereas hyperactive/impulsive, ODD, and autism symptom ratings did not differ significantly. Likewise, inattention ratings were significantly higher in both the low reading and low math achievement test groups in the child community sample. These findings point to the importance of an attention deficit in negatively affecting academic ability as measured by individually administered standardized achievement tests. Therefore, an attention deficit is negatively associated with both academic ability (as measured by individually administered achievement tests) and with academic impairment ratings reflecting academic functioning (difficulty learning even when tries hard, underachieving, low grades, and trouble with reading, writing, or math).

Correlational analyses showed that ratings of inattention were more strongly associated with academic impairment ratings than with autism and ODD ratings in all three samples (referred, child community, and adolescent community), whereas hyperactivity/impulsivity ratings were more strongly associated with ODD than with autism and academic impairment ratings. Across samples, inattention was a significant unique correlate of academic impairment ratings and low reading and math achievement test scores, whereas hyperactivity/impulsivity was a unique correlate of ODD. These findings highlight the importance of addressing inattention to potentially improve learning and academic functioning and addressing hyperactive/impulsive symptoms to reduce ODD symptoms and behavior problems.

### Clinical and Research Implications

Given high symptom co-occurrence, clinicians must evaluate for ADHD, autism, ODD, and academic impairment when one is present. Failing to do so can significantly affect diagnostic conclusions, treatment planning, and outcome expectations, thereby risking the oversight of treatable conditions that may exacerbate one another. The co-occurrence of these conditions increases adverse outcomes and functional impairment for children with autism, ADHD, and/or ODD (Connor et al., 2010; Gadow et al., 2008; Joshi et al., 2017; Rong et al., 2021; Waschbusch, 2002). Costs of not evaluating potential comorbidity at the time of the initial referral are significant. Diagnoses may be delayed and treatment denied.

Early identification of autism is critical so that children can receive evidence-based services, such as applied behavior analysis, which research shows is most effective when provided during the preschool years rather than later (Fenske et al., 1985; Harris & Handleman, 2000). Further, stimulant medication used to treat ADHD symptoms is effective in preschool as well as school-age children (Mayes et al., 1994). Unfortunately, 41% of 6- to 17-year-olds who had autism and ADHD were not receiving medication or counseling for their ADHD, in contrast to 24% of youth with ADHD and no autism in a study by Joshi et al. (2017). Specifically, 44% with autism and ADHD were treated with medication in contrast to 70% with ADHD and no autism.

Placebo-controlled studies in children with autism, ADHD, and/or ODD demonstrate that medication can significantly reduce symptoms of these disorders and improve behavior (Abikoff et al., 2004; Arnold et al., 2006; Connor et al., 2010; Evans et al., 2001; Frazier et al., 2010; Ghuman et al., 2009; Handen et al., 2000, 2008; Harfterkamp et al., 2012, 2014; Jaselskis et al., 1992; McCracken et al., 2002; McDougle et al., 2005; MTA Cooperative Group, 1999; Nagaraj et al., 2006; Pandina et al., 2007; Politte et al., 2018; Scahill et al., 2015; van der Oord et al., 2007; Waxmonsky et al., 2008; Wilens et al., 2013). Medication can also improve academic performance and achievement test scores (Arnold et al., 2020; Baweja et al., 2015; Evans et al., 2001; Jangmo et al., 2019; MTA Cooperative Group, 1999; Scheffler et al., 2009). Nonpharmacological interventions (e.g., applied behavior analysis/behavior therapy) are also effective in decreasing oppositional behavior in children with autism, ADHD, and/or ODD (Brosnan & Healy, 2011; Connor et al., 2010; Fabiano et al., 2009; Frazier et al., 2010; Matson et al., 1996; MTA Cooperative Group, 1999; Waxmonsky et al., 2008).

Specialty clinics should not limit assessments to only one disorder. Many children with autism are initially diagnosed only with ADHD, delaying an autism diagnosis and treatment for 2–5 years or more (Kentrou et al., 2019; Miodovnik et al., 2015; Stevens et al., 2016). Likewise, ADHD is often undiagnosed in children with autism. In fact, the DSM-IV did not permit an ADHD diagnosis if autism was present, thus denying children effective ADHD interventions. Further, ADHD must be considered in children referred for learning problems because most children with learning problems have ADHD, especially an attention deficit, and because pharmacologic and behavioral interventions to treat ADHD can improve academic performance. If not identified, treatment is denied.

Many studies and reviews use prevalence rates based on children identified with ADHD, autism, ODD, or academic impairment when not all children have actually been professionally evaluated for these disorders, thereby underestimating prevalence rates. In two large surveys, only 50% and 47% of children who were significantly elevated on parent ADHD ratings had been evaluated and diagnosed with ADHD (Barkley, 2013; Burns & Becker, 2021). Evaluating all children to rule in or rule out specific disorders is essential in order to obtain accurate prevalence data. The frequency of ADHD in children with autism found in our study is similar to that reported in other studies in which all children were evaluated (e.g., Joshi et al., 2017). However, it is predictably higher than that reported in studies or reviews in which not all children were assessed for ADHD (e.g., Rong et al., 2021).

Research studies also often do not assess children for both ADHD and autism, so findings for children with ADHD may be attributed only to ADHD and findings for autism may be attributed only to autism when findings may actually represent the presence of the other or both. Our findings add to previous studies showing important differences between ADHD-C and ADHD-I. Many studies do not distinguish between ADHD-C and ADHD-I and treat ADHD as a unitary diagnosis.

This study shows that the co-occurrence of symptoms of ADHD, autism, ODD, and academic impairment is fundamental to how these conditions appear in both referred and community youth. The findings suggest that inattention and hyperactivity/impulsivity are separate pathways to academic and behavioral problems, respectively. These results explain why academic challenges continue across different ADHD types and why oppositional behavior is more common in ADHD-C. Significantly, the findings reveal important gaps, especially the under-recognition of ADHD and ODD in autism, that may contribute to delays in providing effective treatment. Overall, the results support the need for comprehensive assessment and treatment strategies rather than single-disorder care models.

### Limitations and Future Research Directions

Although our referred and population-based community samples are representative of the study’s geographic region, the samples are not nationally representative. Our findings need to be replicated in other geographic regions and clinical settings, especially with more racially diverse samples. Further, our clinical sample comprised children specifically referred because of behavior problems and/or possible autism or ADHD, yielding high ADHD and autism frequencies. Our study focused on four of the most common presenting problems for referred children. Future studies should also analyze other domains, such as anxiety, depression, and conduct problems. Children in the population-based community samples did not undergo professional diagnostic evaluations, unlike the referred children. Most population-based community studies, like ours, rely on parent ratings and not rigorous professional diagnoses, which is an obvious limitation. Data from multiple informants (mother, father, teacher, self) should be included in future research analyses. The influence of variables such as demographics, intervention history, educational supports, age, and referral context on the co-occurrence of ADHD, autism, ODD, and academic impairment symptoms needs to be considered. Additionally, our study describes ADHD-C and ADHD-I and symptom co-occurrence at the time of evaluation and is not a longitudinal study investigating ADHD subtype stability, which needs to be examined in future studies.

## Conclusions

Diagnostic symptom co-occurrence was high in referred and community youth. For youth in the ADHD/autism sample, 90% had two or more diagnoses (ADHD, autism, ODD, or academic impairment). In the community samples, two or more elevated symptom ratings in these areas was as common as only one. Overall, most youth with autism symptoms had ADHD-C and ODD symptoms, most youth with ADHD-C had ODD symptoms, and most youth with ADHD-C and ADHD-I symptoms had academic impairment symptoms. In all three samples, the largest correlations with academic impairment ratings were with inattention ratings and the largest correlations with ODD were with hyperactivity/impulsivity ratings. Inattention symptoms were significantly higher in youth with low reading and math achievement test scores. The high prevalence of academic impairment in both ADHD-C and ADHD-I is likely because both subtypes have inattention as a component. Our study findings have important implications for assessment, intervention, and research. When symptoms of one condition are present, likely co-occurring diagnoses should be ruled in or out. Broad band rating scales assessing multiple areas of psychopathology are essential and should be supplemented with diagnostic scales for disorders such as autism that may not be included in broad band instruments. As noted above, research indicates that treating inattention may improve academic performance and treating hyperactivity/impulsivity may alleviate symptoms of ODD and behavior problems. Taken together, these findings indicate that co-occurrence among ADHD, autism, ODD, and academic impairment is fundamental to how these conditions manifest and interact. Models of assessment, intervention, and research that do not explicitly account for this reality risk misattributing symptoms, delaying treatment, and reducing the effectiveness of care.

## Data Availability

Data are not available due to patient confidentiality.
